# An integrated workflow combining machine learning and wavelet transform for automated characterization of heterogeneous groundwater systems

**DOI:** 10.1038/s41598-025-89410-5

**Published:** 2025-02-10

**Authors:** Musaab A. A. Mohammed, Norbert P. Szabó, Abdelrhim Eltijani, Péter Szűcs

**Affiliations:** 1 Faculty of Earth and Environmental Sciences and Engineering, University of Miskolc, Miskolc, Egyetemváros, 3515 Hungary; 2 National Laboratory for Water Science and Water Security, Institute of Water Resources and Environmental Management, University of Miskolc, Miskolc, Hungary; 3 College of Petroleum Geology and Minerals, University of Bahri, Khartoum, Sudan

**Keywords:** Pannonian Basin, Debrecen, Artificial neural networks, Self-organizing map, Wireline log, Environmental sciences, Hydrology

## Abstract

Groundwater aquifers are complex systems that require accurate lithological and hydrogeological characterization for effective development and management. Traditional methods, such as core analysis and pumping tests provide precise results but are expensive, time-consuming, and impractical for large-scale investigations. Geophysical well logging data offers an efficient and continuous alternative, though manual interpretation of well logs can be challenging and may result in ambiguous outcomes. This research introduces an automated approach using machine learning and signal processing techniques to enhance the aquifer characterization, focusing on the Quaternary system in the Debrecen area, Eastern Hungary. The proposed methodology is initiated with the imputation of missing deep resistivity logs from spontaneous potential, natural gamma ray, and medium resistivity logs utilizing a gated recurrent unit (GRU) neural network. This preprocessing step significantly improved the data quality for subsequent analyses. Self-organizing maps (SOMs) are then applied to the preprocessed well logs to map the distribution of the lithological units across the groundwater system. Considering the mathematical and geological aspects, the SOMs delineated three primary lithological units: shale, shaly sand, and sand and gravel which aligned closely with drilling data. Continuous wavelet transform analysis further refined the mapping of lithological and hydrostratigraphical boundaries. The integrated methods effectively mapped the subsurface aquifer generating a 3D lithological model that simplifies the aquifer into four major hydrostratigraphical zones. The delineated lithology aligned closely with the deterministically estimated shale volume and permeability, revealing higher permeability and lower shale volume in the sandy and gravelly layers. This model provides a robust foundation for groundwater flow and contaminant transport modeling and can be extended to other regions for improved aquifer management and development.

## Introduction

The effectiveness of groundwater development programs largely depends on the accurate identification and delineation of the aquifers. The permeability within these aquifers is influenced by the geological properties of the sediments, such as their depositional environment and mineral composition^1^. As permeability governs groundwater movement, recharge, and storage capacity, a thorough understanding of the rock characteristics is essential during the initial phases of groundwater development^2^. Core samples are frequently employed to obtain detailed lithologic and petrophysical data. While core analysis offers high-resolution, precise information on rock properties such as porosity and permeability, the process is often costly and time-intensive^3,4^. Consequently, there is an increasing demand for more efficient techniques that can either supplement or replace traditional core sampling while still offering reliable characterization of subsurface conditions. In recent years, geophysical well logs have been used in groundwater studies due to their ability to provide high-resolution, in situ measurements of the subsurface^5–9^. These continuous records of physical properties along boreholes enable a detailed identification of lithological units, delineate aquifer boundaries, and estimate key hydrogeological parameters.

A persistent challenge in aquifer characterization using well logs is the frequent occurrence of incomplete well logs^10^. Petrophysicists and hydrogeologists often encounter situations where certain types of logs are unavailable due to technical or budgetary constraints. These data gaps can significantly hinder the accurate interpretation of subsurface conditions and limit the effectiveness of subsequent analyses^11^. Traditional methods of dealing with missing data, such as simple interpolation or exclusion of incomplete datasets, may lead to the loss of valuable information and introduce biases in the interpretation. To address this issue, machine learning (ML) models such as k-nearest neighbor (KNN), support vector machine (SVM), and random forest (RF) have been suggested to identify complex relationships between different types of well logs and use this understanding to generate synthetic data for missing log types^12–15^. However, these methods are limited by their inability to capture complex spatial dependencies inherent in well log data, particularly in cases where the relationship between the well logs is non-linear. On the other hand, deep neural networks (DNNs), have demonstrated significant advantages over traditional ML models in the imputation of missing well log data^16–19^. Among these, recurrent neural networks (RNN) and gated recurrent units (GRU) are specifically designed to process sequential data^17,20^. GRU is a simplified variant of the long short-term memory (LSTM) network, retaining similar functionality but with fewer parameters, which makes it less prone to overfitting when dealing with smaller datasets. Unlike other RNN, which uses separate forget and input gates, GRU combines these into a single update gate, reducing computational complexity while maintaining comparable accuracy for many applications. Previous studies have demonstrated GRU’s efficacy in handling geophysical data. For example, Antariska et al.^19^, showed that GRU models achieved comparable performance to LSTM and Bi-LSTM for sequential data tasks but required less training time and memory resources.

The identification of rock types and formation boundaries from the complete well log data presents a significant challenge in subsurface characterization. Log analysts may put different criteria to identify the same lithology and layer boundary; therefore, the consistency of final results is subject to the individual choice of suitable criteria that may lead to ambiguous results^21^. Consequently, there is a growing need for the development and implementation of automated techniques to enhance geological characterization^22,23^. Various multivariate statistical and machine learning methods have been applied in rock typing problems^5,23–28^. For instance, Puskarczyk^29^ used cluster analysis, factor analysis, and neural networks for the determination of the lithological units from well logs. Szabó et al.^30^, used a special cluster analysis technique for rock type identification in gas-bearing formation. Mohammed et al.^31^, used factor analysis for the estimation of shale content in groundwater formation. Liu and Liu^32^ combined different deep neural networks for lithological classification. Self-organizing maps (SOMs) offer distinct advantages in rock typing over the multivariate statistical and unsupervised learning methods, as it is primarily have the ability to handle non-linear relationships and high-dimensional data^33,34^. Furthermore, SOMs do not require pre-labeled training data such as in supervised learning techniques, which is particularly beneficial where core data is sparse or unavailable^23^. Several studies appeared in the literature on using SOM for solving rock typing problems and lithology mapping^26,35–37^.

In addition to SOM, signal processing techniques such as Fourier analysis^38,39^, fractal analysis^40^, and Wash transform^41^, have been used in detecting formation boundaries and characterizing lithological cyclicity. The heterogeneous nature of subsurface geological structures shows distinctive frequency signatures, that need a thorough examination of their spectral patterns. While conventional signal analysis can effectively isolate specific frequency components within a given signal, they are inherently limited in their capacity to pinpoint the depths at which these frequencies manifest^42^. To overcome this limitation, more sophisticated analytical approaches capable of simultaneously resolving both spatial and spectral information are required. In this context, continuous wavelet transform (CWT) analysis has emerged, that is capable of dissecting complex geophysical signals^8,43–46^. For example, Sun et al.^47^, combined wavelet transform techniques with adversarial learning to address the complex task of cross-well lithology identification. Liu et al.^48^, enhanced the mapping of the stratigraphic sequences by employing the wavelet transform on the gamma-ray log. Their study indicated that the sequence cycles obtained from CWT showed high compatibility with those from core analysis.

Machine learning and signal processing techniques have been extensively employed in analyzing geophysical data for lithological characterization of petroleum and gas reservoirs. While these methods have also been widely used to address various hydrogeological challenges, their application to hydrogeological structure identification using geophysical data remains relatively underexplored. Groundwater aquifers often exhibit greater complexity than petroleum reservoirs due to heterogeneous lithology, dynamic recharge processes, and permeability variations. This complexity necessitates the development of more robust methodologies capable of effectively analyzing and predicting hydrogeological parameters. The complexity of groundwater systems calls for more robust methodologies that can effectively analyze and predict hydrogeological parameters. This research aims to develop a comprehensive methodology for the identification and characterization of heterogeneous groundwater systems in the Debrecen area, Hungary. The proposed methodology integrates deep learning, unsupervised learning, wavelet transform analysis to enhance the interpretation of wireline logs and improve the accuracy and efficiency of hydrostratigraphical analysis for a better understanding of groundwater dynamics in the study area. The findings of this study have implications not only for the sustainable management of groundwater resources in the Debrecen area but also for the broader application in the characterization of heterogeneous groundwater systems.

## Study area

**Fig. 1:**
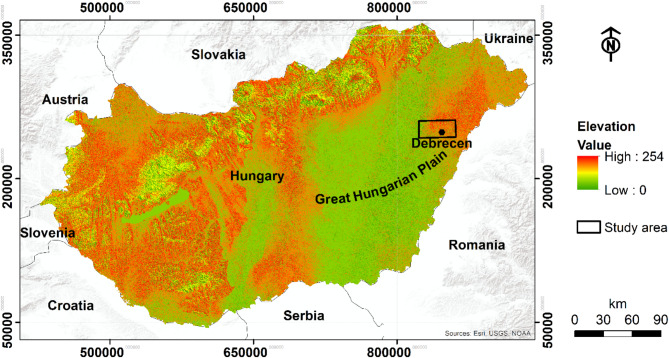
Geographical location of the study area, including elevation information and coordinates based on the EOV (Egységes Országos Vetületi) Hungarian coordinate system. The map is created with ArcGIS Desktop v. 10.8 ^51^.

The study area is centered around Debrecen city in Eastern Hungary which encompasses 650 km^2^ (Fig. 1). This area is part of the Great Hungarian Plain (GHP) that is shaped by tectonic activities, erosion, and continuous deposition processes^49^. These geological forces have resulted in a varied topography within the study site, with elevations ranging from 81 to 164 m above sea level (a.s.l). The climate in the GHP is continental, characterized by average annual temperatures between 10° and 11° C. The precipitation patterns exhibit seasonal variations, with yearly totals averaging between 550 and 600 mm. However, the region experiences a water deficit, as the potential evapotranspiration surpasses the rainfall, reaching up to 700 mm annually^50^.

**Fig. 2:**
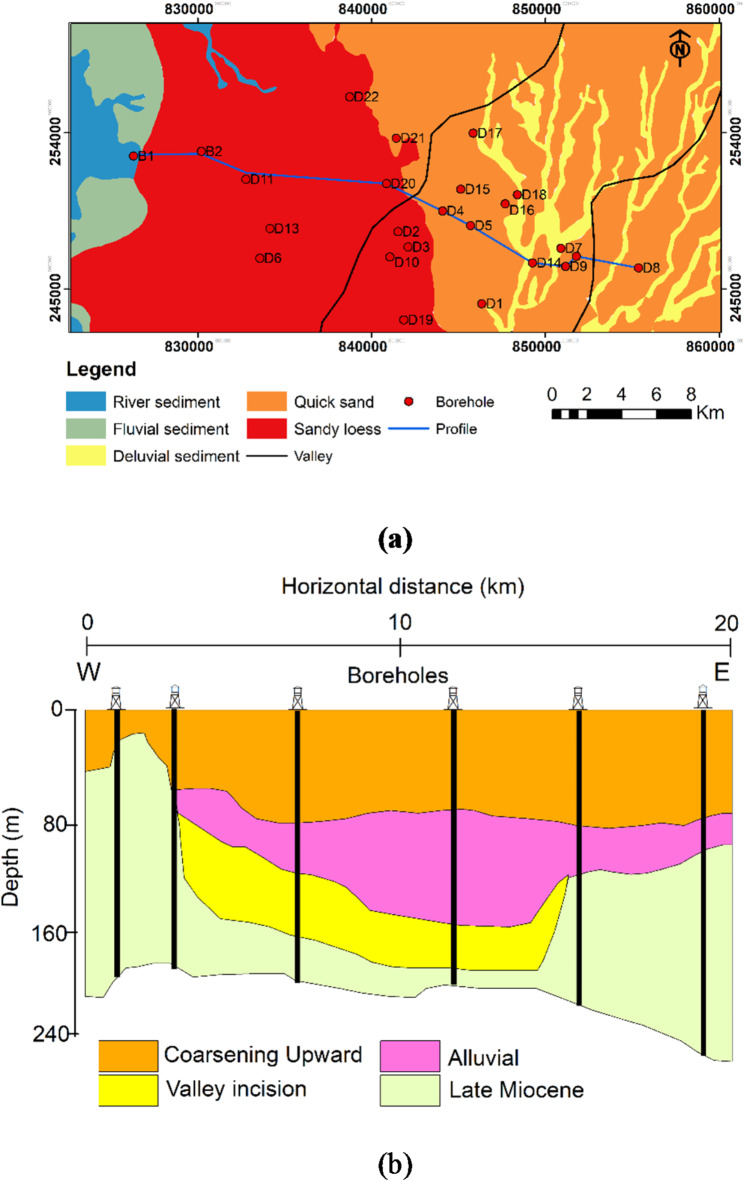
(**a**) Surface geology of the Debrecen area modified after^56^. The map also shows the distribution of the boreholes and profile. The black lines of the valley represent the eastern and western boundary of the valley in which the incised valley deposits are observed (**b**) Hydrostratigraphical units within the Quaternary aquifer system in the study area modified after Carpio^53^.

The surface geology is primarily dominated by Quaternary deposits of Pleistocene age. These deposits are predominantly composed of fluvial sediments, including river gravels and sandy loess (Fig. 2a). Quaternary deposits in the study area can be further subdivided into Upper, Middle, and Lower Pleistocene units. The Lower and Upper Pleistocene sub-units predominantly comprise river and overbank sediments, while the Middle Pleistocene unit is characterized by fine-grained fluvial-lacustrine deposits^52^. This study investigates the Quaternary system that boasts a permeability exceeding 1000 mD. A recent investigation by^53^ examined the upper 280 m of the Great Plain Aquifer revealing that the Pre-Quaternary and Quaternary sequences are the key hydrostratigraphic components (Fig. 2b). The Pre-Quaternary sequence (the Late Miocene unit), is made up of silt occasionally intercalated with fine sands. The Quaternary sequence is divided into three hydrostratigraphic units including; incised valley, alluvial, and coarsening upward units. The incised valley unit is characterized by elongated ribbon-shaped sand and gravel with less clay content oriented northeast-southwest, this unit is overlayed by the sandy alluvial unit; the alluvial unit is characterized by horizontally heterogeneous sand body intercalated with silty-clay deposits. Finally, the coarsening upward unit presents diverse interbedding of clay, silt, and sand layers. The hydraulic conductivity of the coarsening-upward unit varied from 0.0001 to 11.5 m/d, with the lowest value observed in the shaly layers. The alluvial unit exhibited lower hydraulic conductivity, ranging from 0.00003 to 6.6 m/d. In contrast, the valley incision deposits had values between 0.1 and 8 m/d. The Late Miocene deposits displayed the widest range, with hydraulic conductivity fluctuating between 0.53 and 15.3 m/d^54^. The primary source of recharge flow within the Great Plain Aquifer is rainwater infiltration influenced by gravity flow^55^.

## Materials and methods

In this study, an integrated methodology combining machine learning and signal processing techniques was developed to improve the lithological and hydrogeological characterization of groundwater aquifers. The approach begins with data preprocessing, where the missing well log is imputed using a GRU neural network. SOM is then employed to cluster and interpret lithological variations, followed by CWT analysis to identify formation boundaries and lithological cyclicity. Finally, deterministic methods are applied to estimate hydrogeological parameters, providing a comprehensive assessment of the subsurface formations. The rationale for this combination lies in taking advantage of the complementary strengths of each method. This integrated approach improves the resolution and accuracy of the characterization of complex groundwater systems while offering better interpretability compared to conventional well-logging analysis.

### Dataset

These methods are employed on the geophysical well logs that include spontaneous potential (SP), natural gamma ray (NGR), medium resistivity (RS), and deep resistivity log (RD) obtained from 24 boreholes. However, the RD log is encountered in a limited number of 4 boreholes (D2, D14, D15, and D22). In the study area, well-logging data is primarily acquired for well-design purposes, where medium and shallow resistivity logs are typically sufficient to address the operational requirements. However, for a detailed estimation of aquifer characteristics, the inclusion of RD logs would indeed provide additional value. RD logs allow for better resolution of deeper layers unaffected by borehole and mud effects, thus offering improved accuracy in characterizing the aquifer’s properties. The logs are recorded at a logging interval of 0.1 m and the depth of the boreholes range from 105 to 198 m. Some boreholes (54%) fully penetrate the entire thickness of the Quaternary formation, However, the remaining boreholes only penetrate the upper hydrostratigraphic units, specifically the coarsening upward and alluvial units. In addition to the borehole data, information about mud resistivity and the resistivity of the formation water was obtained to facilitate the accurate estimation of petrophysical parameters. The mud resistivity helps correct the borehole environment effects, while the formation water resistivity is crucial for calculating water saturation and understanding the pore fluid properties.

### Gated recurrent unit (GRU) neural network

**Fig. 3:**
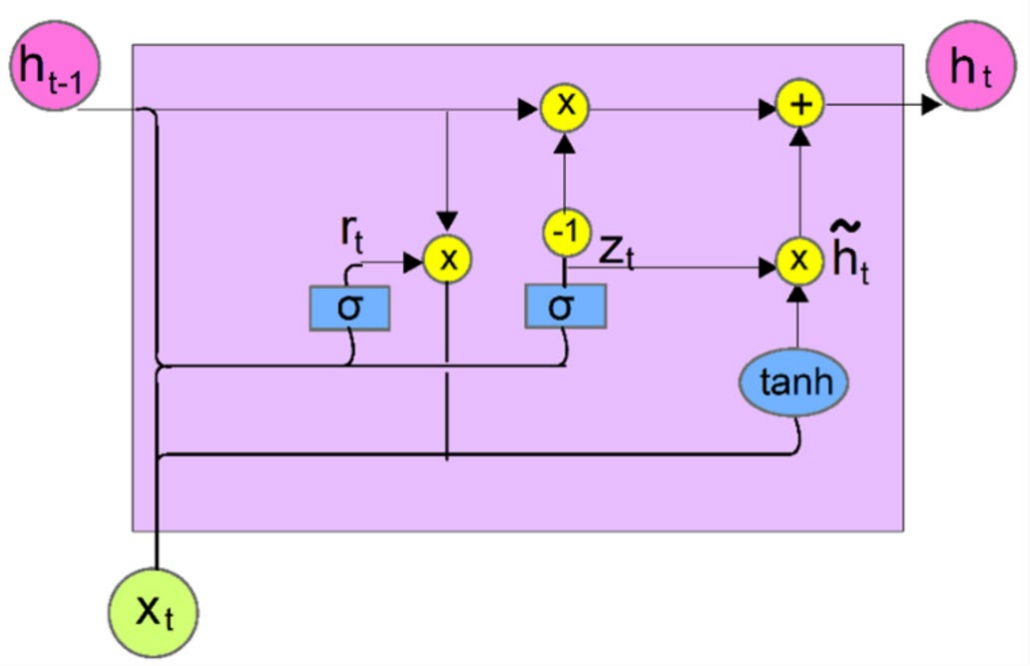
The structure of the gated recurrent unit (GRU) illustrating key components: x_t_​ (input), h_t−1_​ (previous hidden state), h_t_ (current hidden state), r_t_​ (reset gate), z_t_​ (update gate). The GRU utilizes sigmoid (σ) and tanh activation functions to balance memory retention and update dynamics through element-wise operations (+, x).

A GRU neural network is trained and utilized to predict missing deep resistivity log (RD) in the dataset, ensuring a continuous and reliable dataset for accurate lithological characterization. GRU is a variant of RNNs specifically designed to capture long-range dependencies within sequential data^57^. The RGU consists of a series of interconnected gated units, including input, forget, and output gates (Fig. 3). This gating mechanism allows the GRU to selectively update and reset its internal state based on the input data and prior knowledge, enabling effective learning of complex relationships^58^. The update gate filters the flow of past information to the future, while the reset gate selectively prunes unnecessary memories. By iteratively adjusting the model parameters through backpropagation and gradient descent optimization, the predictive performance of the GRU model is optimized and the trained GRU model is then utilized for the imputation of missing RD log. The update gate ($\:{z}_{t}$) is represented by Eq. 1 while the reset gate ($\:{r}_{t}$) by Eq. 2. The output of the hidden layer ($\check{{\text{h}}}_{t}$) is expressed by Eq. 3 while the hidden state update or the output ($\:{h}_{t}$) is calculated by Eq. 4.

1 $$\:{z}_{t}=\:\sigma\:\left({W}_{z}*{x}_{t}+{W}_{z}*{h}_{t-1}+{b}_{z}\right)$$

2 $$\:{r}_{t}=\:\sigma\:\left({W}_{r}*{x}_{t}+{W}_{r}*{h}_{t-1}+{b}_{r}\right)$$

3 $$\check{{\text{h}}}_{t} =tanh\left({W}_{h}*{x}_{t}+{W}_{hc}*{h}_{t-1}+{b}_{h}\right)$$

4 $$h_{t} = \left( {1 - z_{t} } \right) \odot h_{{t - 1}} + z_{t} \odot \: \check{{\text{h}}}_{t}$$

Where $\:{x}_{t}$ is the input at depth point t, $\:{h}_{t-1}$is the output of the previous neuron σ denotes the sigmoid activation function, $\:tanh$ is the hyperbolic tangent activation function, $\:W$ is the weight matrices, b denotes the bias vectors, and $\odot$ is the Hadamard product (Element-wise product).

The GRU model was implemented using Python version 3.7 within the Keras and TensorFlow frameworks. The GRU model used in this study was configured with two hidden layers consisting of 100 and 50 units, respectively. The number of hidden layers and neurons in each layer were determined experimentally through a trial and error approach. For this study, we evaluated architectures with 1–3 hidden layers and varying neuron counts (e.g., 16, 32, 64, and 128 neurons per layer) to balance model complexity and predictive accuracy. The Adam optimizer was employed to optimize the model’s weights. Additionally, the rectified linear unit (ReLU) activation function was utilized to introduce non-linearity into the model. The learning rate was chosen based on experimentation, with initial values ranging from 0.005 to 0.01, evaluated to optimize convergence speed and minimize loss. A value of 0.002 was found to yield the lowest validation error while maintaining stable training dynamics.

To evaluate the performance of the GRU model in imputing missing well log data, three key performance metrics were used: root mean square error (RMSE) (Eq. 5), mean absolute error (MAE) (Eq. 6), and the coefficient of determination (R^2^) (Eq. 7). Together, these metrics offered a comprehensive evaluation of the model accuracy and overall predictive performance.

5 $$\:\text{R}\text{M}\text{S}\text{E}=\frac{1}{n}\sqrt{\sum\:_{i=1}^{n}{\left({x}_{i}-{y}_{i}\right)}^{2}}$$

6 $$\:\text{M}\text{A}\text{E}=\frac{1}{n}\:\sum\:_{i=1}^{n}\:\left|{x}_{i}\:-{y}_{i}\right|$$

7 $$\:{\text{R}}^{2} = \left( {\frac{{\left[ {\sum {\:_{{i = 1}}^{n} } \:\left( {y_{i} - \overline{y} } \right)\:\left( {x_{i} - \overline{x} } \right)} \right]^{2} }}{{\sqrt {\sum {\:_{{i = 1}}^{n} } \:\left( {y_{i} - \overline{y} } \right)^{2} \sum {\:_{{i = 1}}^{n} } \left( {x_{i} - \overline{x} } \right)^{2} \:} }}} \right)^{2}$$

Where n is the total number of the data points, y and x represent the predicted and actual values, and ӯ and x̄ are the mean values of the predicted and actual values, respectively.

### Self-organizing maps

**Fig. 4:**
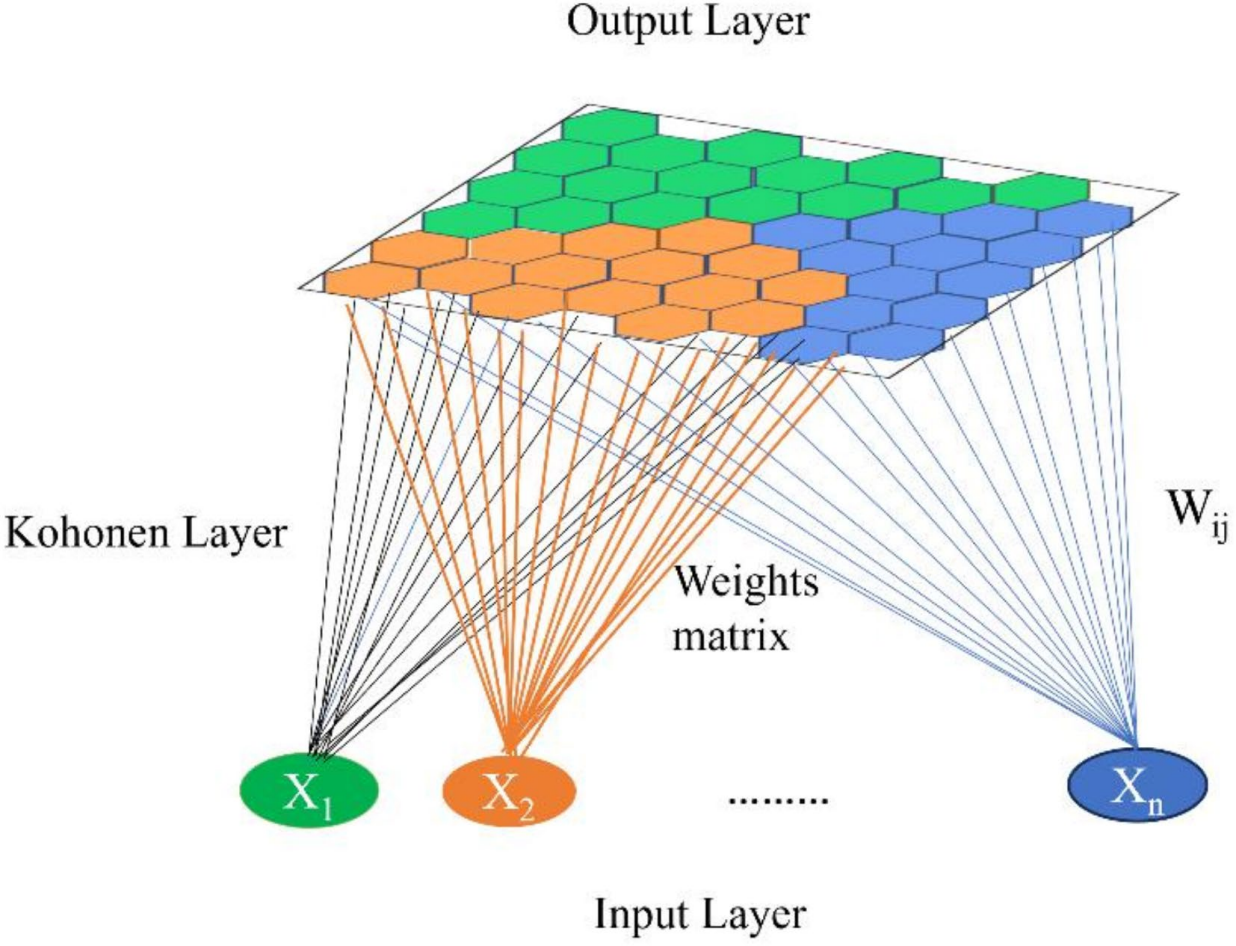
The structure of the SOMs in which X is the data raw vector at a specific depth for multiple well logs that provided weights (W) based on the distance from the best matching neuron.

Self-organizing maps (SOMs), a type of unsupervised artificial neural network, were employed in this study to enhance the lithological interpretation of well-log data. The SOM algorithm, initially proposed by^59^, operates on the principle of competitive learning. In the recent application, each neuron in the SOM represents a potential lithological class, characterized by a weight vector (W_ij_) of the same dimension as the input depth point of all logs simultaneously (X_1_, X_2_, … X_n_) (Fig. 4). The training process involves iteratively presenting well log data to the network, identifying the best matching unit (BMU) – the neuron whose weight vector is most similar to the input data – and updating the weights of the BMU and its neighboring neurons. This process results in a self-organized map where similar lithological classes are represented by neurons in close proximity on the two-dimensional output grid.

In this study, SOMs are conducted in MATLAB framework and applied to both the raw and preprocessed well-log data. The input vectors for the SOM included measurements from multiple logging tools, such as SP, NGR, and RS, along with the synthetic RD log generated by our GRU neural network. The choice of SOM architecture, including the number of neurons and the grid topology, was determined through a series of experiments to optimize the trade-off between resolution and generalization. Hexagonal grid topology is employed, which provides a more natural representation of neighborhood relationships compared to a rectangular grid. The number of neurons (n) was determined using the common heuristic equation of n= √N where N is the number of data points. The learning rate was initialized at 0.1 and gradually decayed during training through 200 epochs to refine the clustering as the SOM converged.

### Wavelet analysis

The continuous wavelet transform (CWT) was employed on the NGR to analyze the cyclicity and spatial variability of lithological characteristics within the Quaternary aquifer system. This technique allows for the simultaneous analysis of time (depth in this case) and frequency content within a signal^60^. In WT, a “mother wavelet” function (*ψ*(*t*)) serves as the basis for generating a family of daughter wavelets through scaling (dilation or compression) and translation (shifting) operations^61^. The choice of the mother wavelet is critical in CWT analysis, as it influences the resolution and interpretability of the results. The daughter wavelets are defined (Eq. 8) as

8 $$\:\psi\:a,\:b\:\left(t\right)\:=\:{a}^{(-1/2)}\:\psi\:\left(\right(t\:-\:b)/a)$$

Where *a* (> 0) is the scale (dilation) of the wavelet, b represents the position (translation), a^(−1/2)^ is the normalization factor ensuring consistent energy across daughter and mother wavelets^62^. The continuous wavelet (CWT) of a signal* s*(*t*) is then calculated (Eq. 9) through the convolution with the analyzing wavelet* ψ*(*t*) as

9 $$\:W(a,\:b)\:=\:\int\:s\left(t\right)\:\psi\:*a,b\left(t\right)\:dt$$

Where ψ*a, b(t) denotes the complex conjugate of the scaled and translated mother wavelet, *a* and *b* represent the scaling and translation parameters, respectively. By comparing the signal to these shifted and scaled wavelets at various scales and positions, CWT provides a two-dimensional representation of the signal’s frequency content across depth^63^. The results of the CWT were visualized using scalograms, which display the magnitude of wavelet coefficients as a function of both scale and depth. These scalograms provide a comprehensive view of the frequency content of the well logs across different spatial scales, enabling the identification of dominant cyclic patterns and their vertical extent within the aquifer system.

### Petrophysical parameters

In this study, petrophysical and hydrogeological parameters including shale volume, effective porosity, and permeability were estimated to characterize the identified lithological interpretations derived from SOM and CWT analyses. The shale volume (V_sh_) is determined using Larionov^64^ formula (Eq. 10) in which the NGR intensity (I) is determined by Schlumberger^65^ formula (Eq. 11). The Larionov equation was selected because it is well-suited for shale volume estimation in both younger and older sedimentary formations, making it applicable to the Quaternary and Miocene deposits in the study area.

10 $$\:{V}_{sh}=0.33\:\left({2}^{2*{I}_{\gamma\:}}-\:1\right)$$

11 $$\:{I}_{\gamma\:}=\:\frac{{GR}_{log}-\:{GR}_{min}}{{GR}_{max}-\:{GR}_{min}}$$

Where GR_min_ is the minimum gamma-ray reading corresponding to a clean, clay-free formation and GR_max_ is the maximum gamma-ray reading corresponding to a fully shaly formation. These were derived directly from the GR logs for each borehole.

The effective porosity in this study was determined (Eq. 12) using porosity values estimated by Archie’s formula^66^ considering the shale content in the groundwater formation^67^. Archie’s equation relates the formation factor (F) to the porosity ($\:{\upphi\:}$) and resistivity of the saturated formations. The F can be calculated using Eq. 12. Accordingly, the Archie porosity can be estimated using Eq. 13. On the other hand, permeability (k) (mD) in this study was estimated based on the effective porosity ($\:{{\upphi\:}}_{\text{e}}$) (Eq. 14) of the aquifer materials using the Jorgensen formula^68^ (Eq. 15).

12 $$\:F=\:\frac{{R}_{0}}{{R}_{w}}$$

13 $$\:F=a\:{\phi\:}^{-m}$$

14 $$\:{\phi\:}_{e}=\phi\:\:*(1-\:{V}_{sh})$$

15 $$\:k=8400\left(\frac{{{\phi\:}_{e}}^{m+1}}{{\left(1-{\phi\:}_{e}\right)}^{2}}\right)$$

Where R_0_ is the resistivity of the formation, R_w_ is the resistivity of water formation which is obtained from the laboratory analysis, a is the tortuosity factor, and m is the cementation factor that ranges from 1.8 to 2.5 but in this study, a value of 2 is accepted to represent the aquifer system. The estimation of aquifer parameters using Archie and Jorgensen methods has limitations in capturing aquifer heterogeneity. These methods work best in low-shale aquifers. Additionally, empirical parameters like tortuosity factor and cementation exponent may not accurately reflect the aquifer’s flow path characteristics. This may lead to inaccuracies however a generalized image of the system can be obtained.

## Results and discussion

### Imputation of RD log

**Fig. 5:**
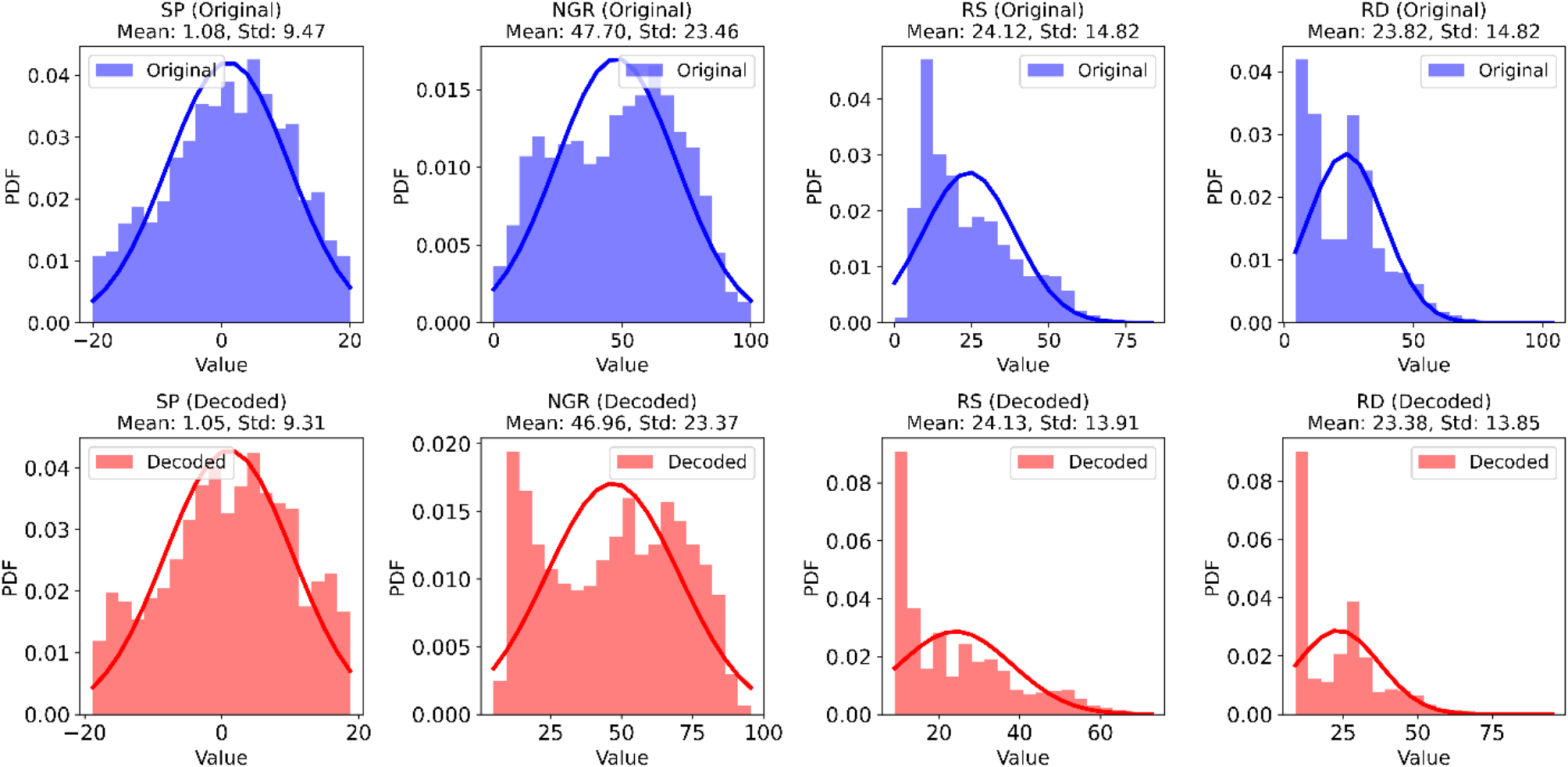
The probability distribution functions and probability density estimate (blue line) for the distribution of the well logs. The RD data is obtained from the 4 boreholes with the complete data.

In Hungary, groundwater well logging typically excludes the RD log, as groundwater well logging is primarily used for well design purposes; however, this log provides insights into the intrinsic properties of subsurface materials beyond the invaded zone, making it indispensable for the accurate estimation of lithological and hydrogeological properties. In this study, the RD log is predicted based on the available SP, NGR, and RS logs using GRU neural network. Initially, the probability density function (PDF) of the input logs was analyzed (Fig. 5) to assess their distribution and variability. The SP logs ranged from − 20 to 20 mV, with a mean value of 1.08 mV and a standard deviation of 9.47, indicating a relatively narrow spread around the mean. The NGR logs, ranging from 0 to 100 API, had a mean of 47.7 API and a higher standard deviation of 23.5, reflecting greater variability in NGR measurements. The resistivity logs exhibited values between 2.2 and 81.2 Ωm, with a mean of 24.12 Ωm and a standard deviation of 14.8. The SP log exhibits an approximately symmetrical distribution, resembling a normal distribution, suggesting relatively balanced variations around a central value. In contrast, the NGR log shows a slightly right-skewed distribution, indicating a tendency toward higher values. The RS log displays a highly right-skewed distribution, with a pronounced tail extending toward higher resistivity values, reflecting substantial variability and possible heterogeneities. Similarly, the RD log also exhibits a right-skewed distribution, though its peak is sharper, and the tail is less pronounced compared to the RS log. These distributions highlight variations in the geological properties captured by each log.

**Fig. 6:**
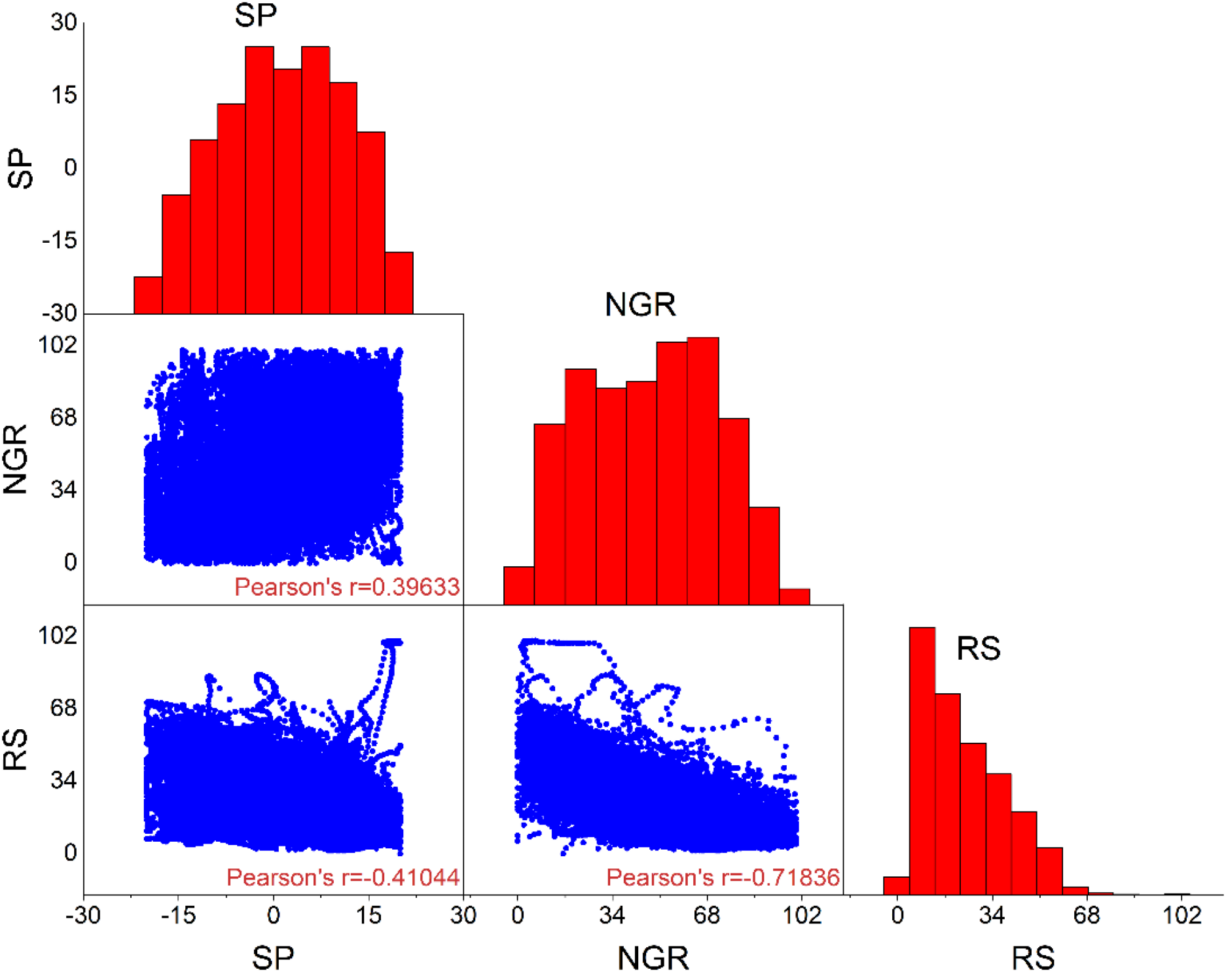
Scatter matrix showing the correlation between the input logs from all boreholes used for prediction of RD log.

In geological formations, well logs often exhibit varying degrees of correlation, depending on the specific lithological and hydrological conditions of the formation. Generally, these logs are not perfectly correlated because they measure different physical properties^9^. Given the moderate to high Pearson^69^ correlation observed between the input logs of all boreholes (Fig. 6), a simple GRU model was chosen as fewer variables need to be considered for accurate predictions. The moderate Pearson correlation between SP and RS (-0.41) and SP and NGR (0.40), and high correlation between RS and NGR (-0.72) indicate that while there are discernible relationships among the logs, they are not so complex as to necessitate highly complex model. A relatively straightforward architecture is well-suited to capture and learn from these moderate correlations without overfitting or requiring excessive computational resources. Consequently, the GRU model was trained on a borehole with a complete log suite (D15 borehole) that is located in the central part of the study area. This borehole is selected to allow the GRU model learning from a representative dataset that captures the typical lithological and geophysical characteristics of the Quaternary aquifer system. The trained model was then applied to other wells across the study area to predict missing RD logs, ensuring consistency and accuracy in the dataset.

**Fig. 7:**
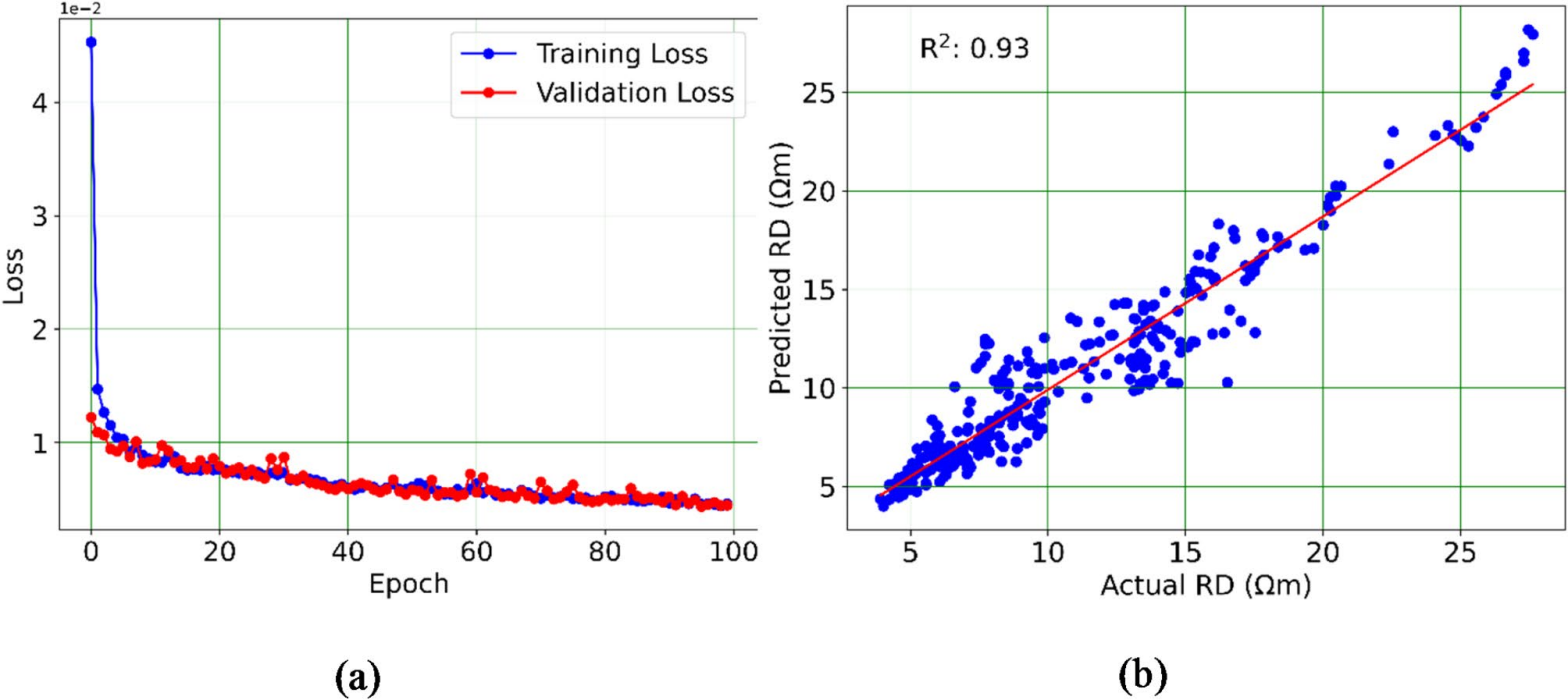
(**a**) Representation of the training and validation for the GRU model throughout the epochs and (**b**) The correlation between the observed and predicted deep resistivity log in borehole D2.

**Fig. 8:**
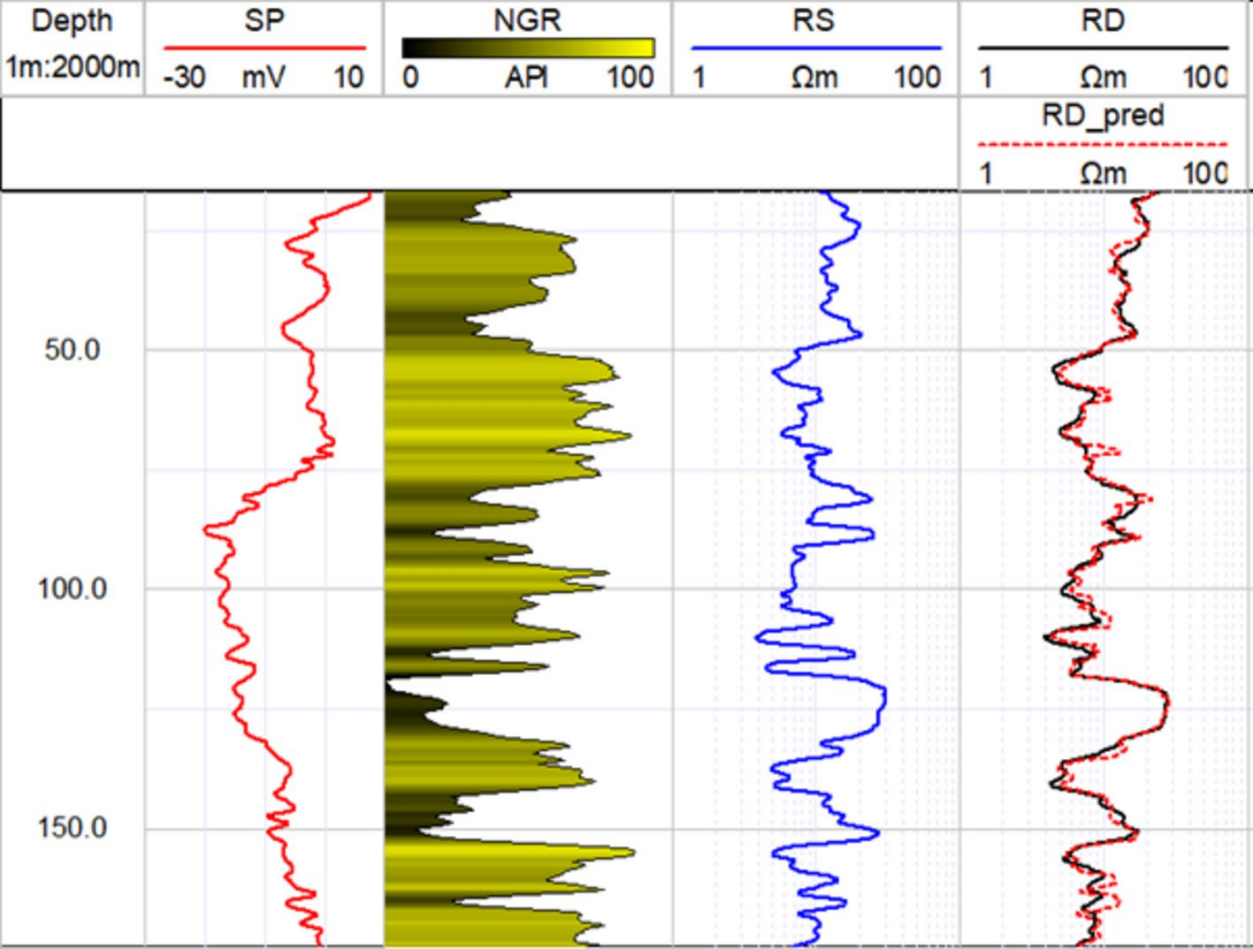
The results of deep resistivity (RD) log prediction using gated recurrent unit model along the D2 borehole based on SP, NGR, and RS logs as input features.

The structure of the GRU model including the number of hidden layers and neurons was determined through experimentation and a trial-and-error approach. Various configurations were tested, however; two hidden layers with 100 and 50 neurons are selected. The GRU model was trained and validated over 100 epochs to ensure robust performance and convergence. The representation of the training and validation is shown in Fig. 7a. The typical loss pattern for training and validation throughout the epochs signals that the model is learning effectively, generalizing well to new data, and not overfitting, which are all positive indicators of model performance. Accordingly, the logs generated with GRU showed a strong correlation with actual RD measurements in validation wells (D2), with an average R-squared value of 0.93 (Fig. 7b), a root mean square error (RMSE) of 0.06 Ωm, and a mean absolute error of 1.07 Ωm. An example of the 1D comparison between the actual and predicted RD log is illustrated in Fig. 8 where a strong correlation is indicated. However, some limitations were observed, particularly in depths where abrupt increases in the log values occurred, as well as in the peaks of the RD log. This indicated that the prediction lacks minimal spatial resolution. However, the overall performance of the GRU network can be considered successful for RD log prediction to be used for further analysis. The use of the GRU neural network significantly outperformed other machine learning methods^10,70,71^, in predicting well-log data. This superior performance is attributed to its advanced architecture, which allows it to better model the sequential nature of well-log data and handle the intricacies of missing values.

### Lithological mapping

#### SOM analysis

**Fig. 9:**
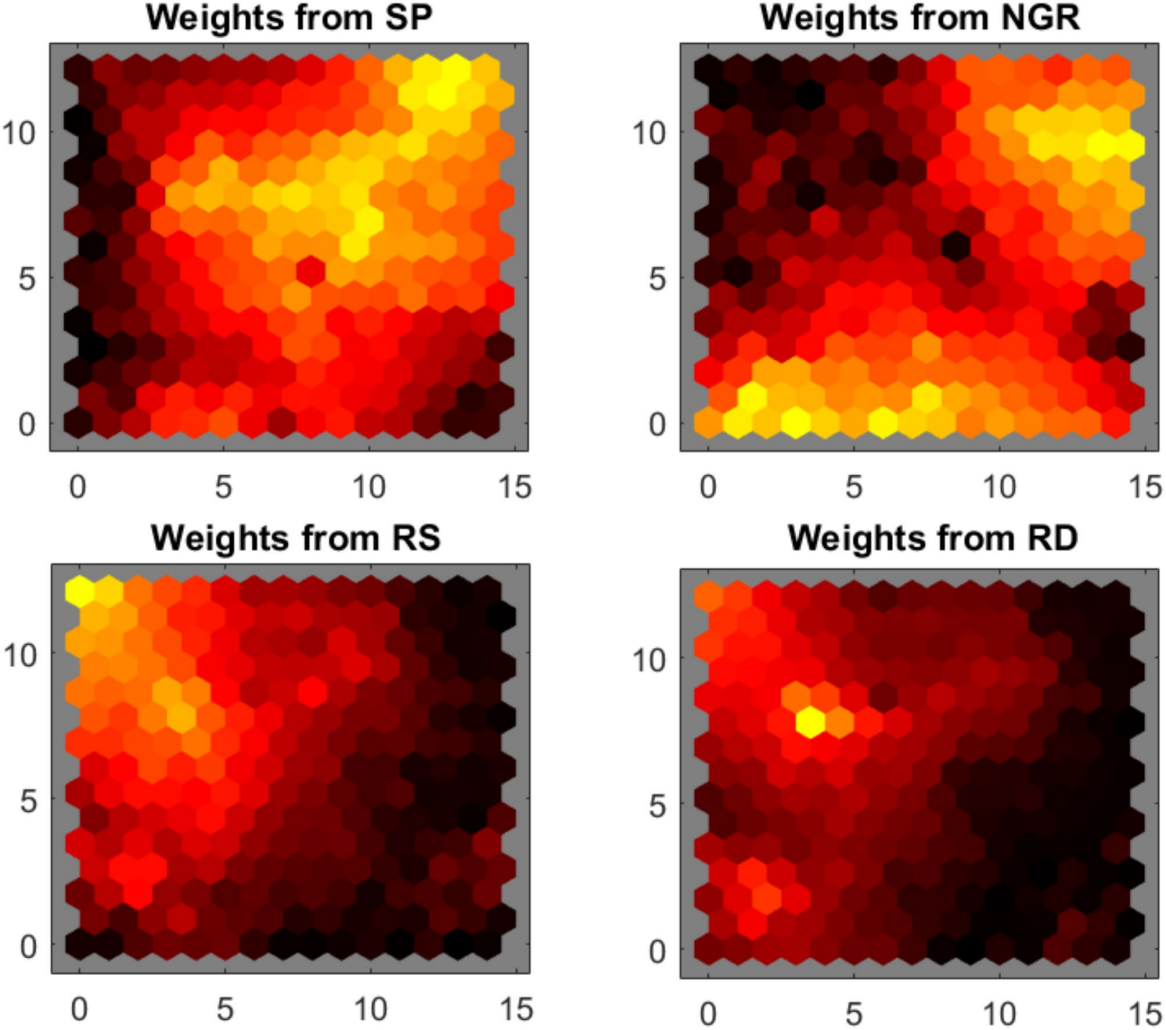
The 2D representation of the weight planes of the SOMs for each well log in all boreholes employed using 225 neurons. The warm color code represents the low values with dark colors (black) and higher values with lighter colors (yellow).

The lithological characteristics of the groundwater system and their spatial distribution were mapped using SOMs. This ML model classifies the well-log data into distinct lithological units by organizing the data into clusters. In general, the more well logs incorporated into the SOM clustering, the more accurate and detailed the lithological mapping becomes, as additional data enhances the ability to distinguish between different lithological units^71^. The application of the SOM involved using the RD log generated through the GRU neural network in addition to the SP, NGR, and RS logs to enhance the accuracy of lithological predictions. Initially, the SOM was performed using 225 neurons to visualize the weight planes of each log to understand their associations and differences. The weight planes provided a visual representation of how each log contributed to the clustering process (Fig. 9). The SOM analysis delineated three primary lithological clusters, visually represented by distinct chromatic categories: yellow, orange, and black. These color designations correspond to specific variations in well log responses, with yellow indicative of elevated NGR corresponding to lower RS and RD in the resistivity weight planes (Fig. 9). This indicates that SOM effectively preserved the topological relationships among the input log parameters, exhibiting consistent patterns and gradual transitions across the two-dimensional map space.

**Fig. 10:**
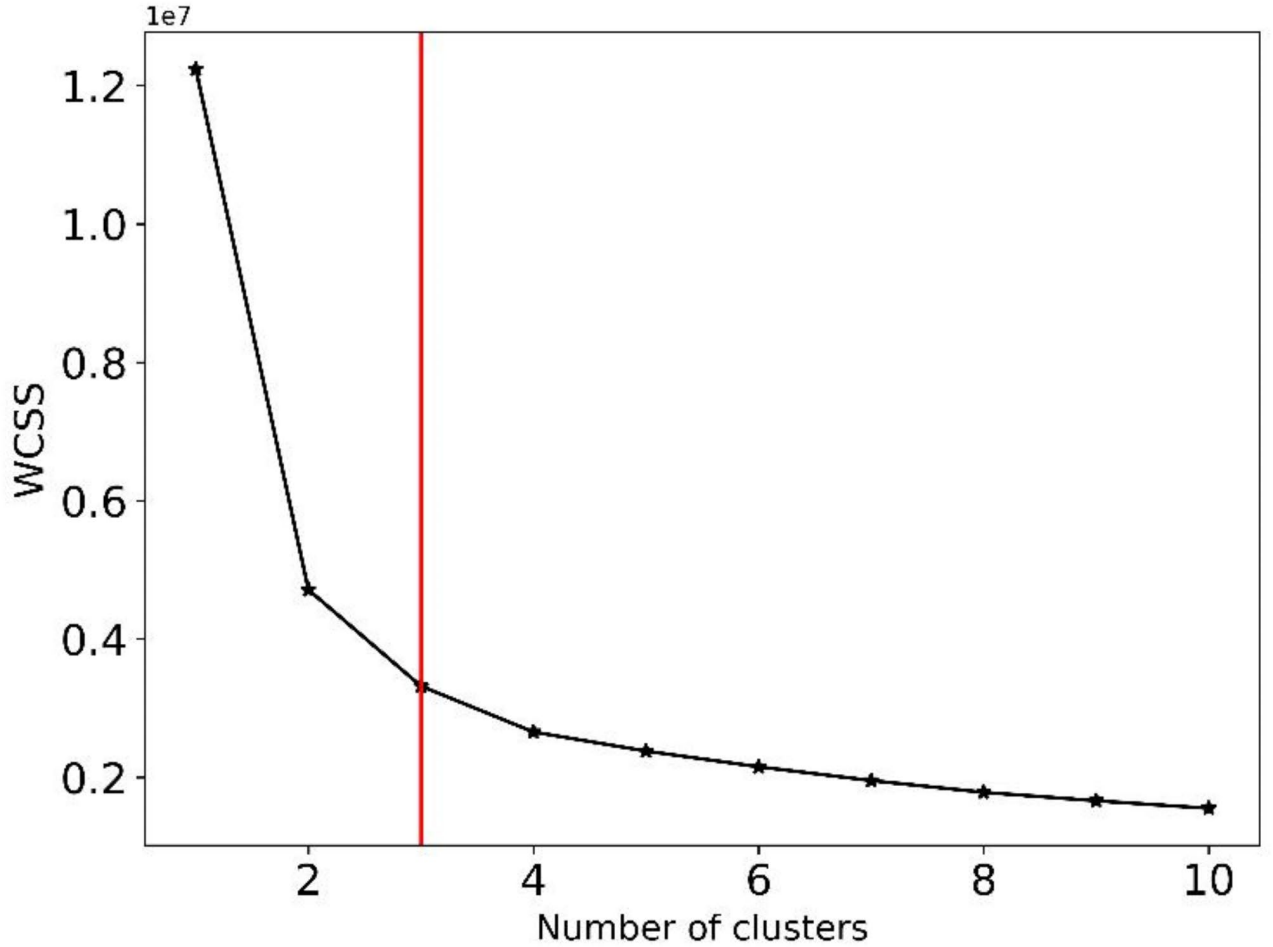
Selection of the optimal number of clusters using the Elbow method.

**Fig. 11:**
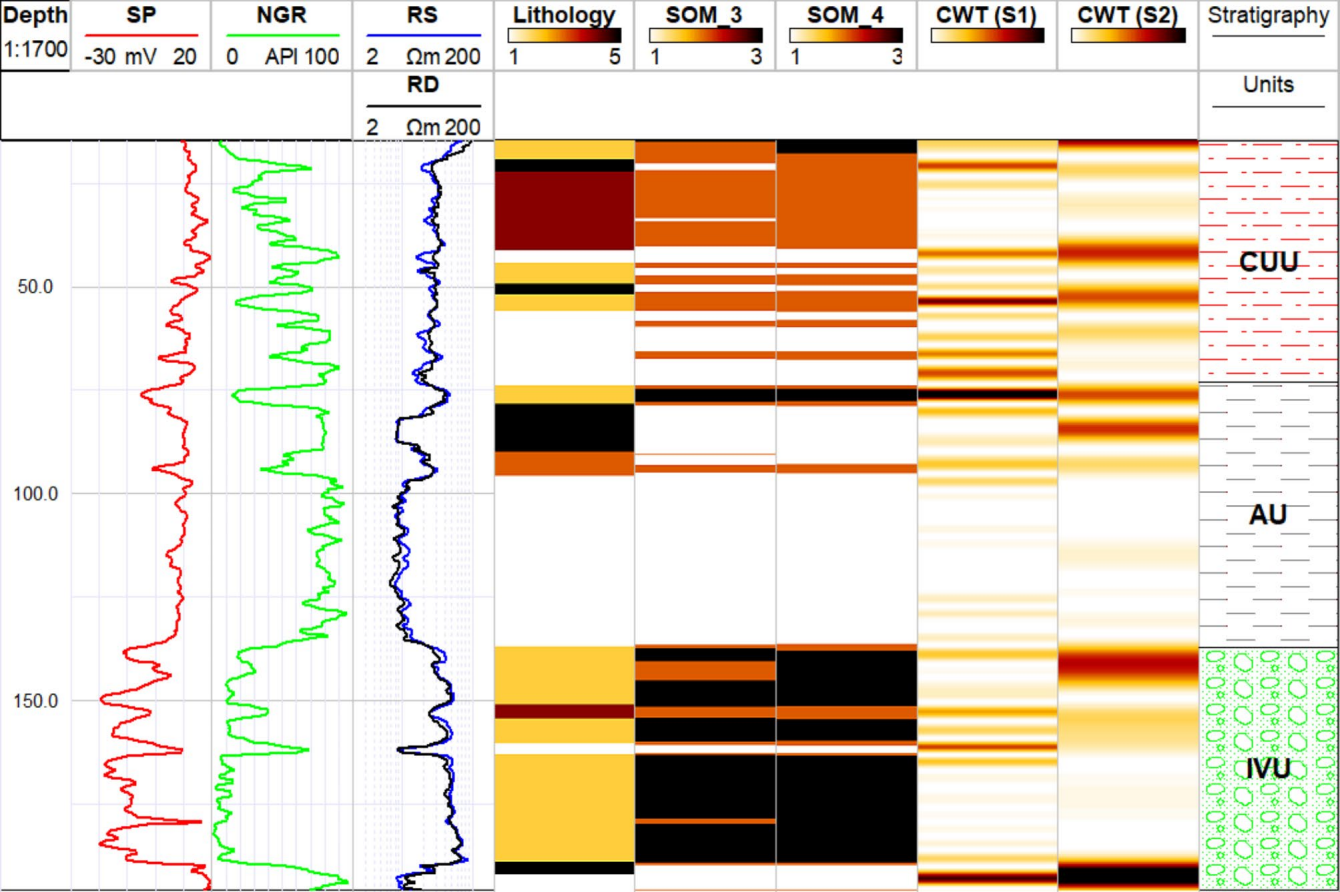
1D representation comparing the core-based lithology (Track 4) to the lithology obtained by analysis of the well logs using SOM (Tracks 5 and 6) and CWT analysis (Track 7 and 8). The SOM_3 represents the lithology obtained using 3 well logs while SOM_4 is the lithology after the imputation of the RD log. CWT is employed in two scales which are fine (S1) and coarse scale (S2).

Following this exploratory phase, the SOM was refined to use 3 neurons, corresponding to 3 lithological clusters. This number of neurons is determined by using the Elbow method, silhouette coefficients^72^, and previous knowledge about the geology of the study area. The Elbow method suggested that the optimal number of clusters is 3 (Fig. 10), as this configuration balanced the variance reduction effectively while providing meaningful clusters^30^. This is also supported by an average silhouette value of 0.50, indicating a good level of cohesion within clusters and separation from neighboring clusters. Additionally, the choice of 3 lithological clusters is grounded in the understanding that the Quaternary sediments in the Great Hungarian Plain (GHP) form a complex fluvial system predominantly consisting of clastic sediments^73^. This system is shaped by varying fluvial transport capacities, produces a diverse range of rock textures, from shale to gravel^74^. The resulting clusters were analyzed in 1D, 2D, and 3Ds as follows: Cluster 1 was characterized by high NGR values and low resistivity values, indicative of shale. Cluster 2 exhibited medium values for both NGR and resistivity, representing shaly sand. Cluster 3 was associated with high resistivity values and low NGR values, corresponding to sand and gravel lithologies (Fig. 11).

In the 1D analysis, SOM-derived lithology was compared with lithology obtained from drilling (Fig. 11), showing a closer agreement when four well logs were used compared to three. When only three well logs were used in the SOM analysis, unrealistic thin layers appeared within thick, continuous layers, leading to an inaccurate representation of the system. Additionally, the boundaries between lithological layers were not accurately mapped, resulting in a less reliable depiction of the lithological units. These limitations were not observed when four logs were used, as the additional data provided a more coherent and realistic mapping of the subsurface lithology. While the SOM clustering identified three distinct lithological units, core lithology analysis revealed five units. This discrepancy is because multiple lithologies from the core data are grouped into a single cluster in the SOM analysis. For example, lithologies such as silty clay, clayey sand, and silty sand identified from drilling are all classified under the shaly sand category (Cluster 2). This suggests that the SOM clustering simplifies complex lithological variations by combining similar lithologies into broader categories, potentially reducing granularity in the interpretation^75^.

**Fig. 12:**
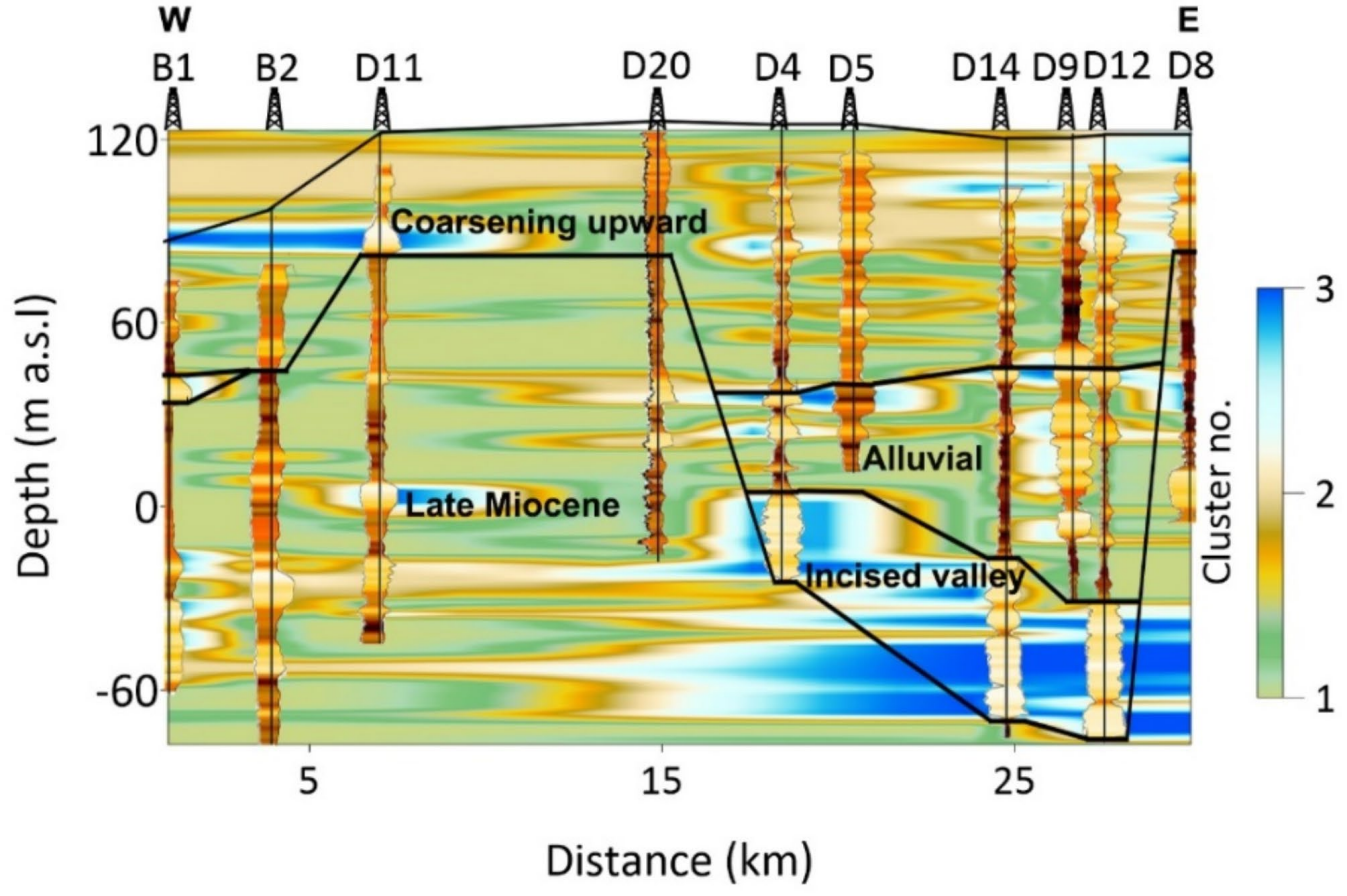
The 2D distribution of the clusters along the profile obtained by SOM using SP, NGR, RS, and RD logs.

Furthermore, 2D analysis of lithological clusters along a profile (see Fig. 2a) is performed to map the lithological variations within the hydrostratigraphical units of the groundwater system (Fig. 12). The 2D analysis results were obtained using kriging interpolation between the data from individual boreholes. The hydrostratigraphical units include the coarsening upward unit (CUU), alluvial unit (AU), incised valley unit (IVU), and Late Miocene unit (LMU). The CUU exhibits a diverse array of lithologies, primarily composed of shaly sand layers (Cluster 2), which are intermittently separated by shaly layers (Cluster 1). Sandy zones (Cluster 3) appear sporadically throughout the CUU, providing localized higher permeability zones. In contrast, the AU is dominated by shaly layers (Cluster 1) with occasional sand bodies showing significant horizontal variation, indicating localized sand deposition. The IVU consists mostly of sand and gravel (Cluster 3), marking it as the most permeable and groundwater-productive zone, although thin layers of shaly sand (Cluster 2) occasionally separate the sand bodies. The LMU is largely composed of shale deposits (Cluster 1), with occasional intercalations of shaly sand (Cluster 2), reflecting a more mixed lithological structure dominated by fine-grained sediments.

**Fig. 13:**
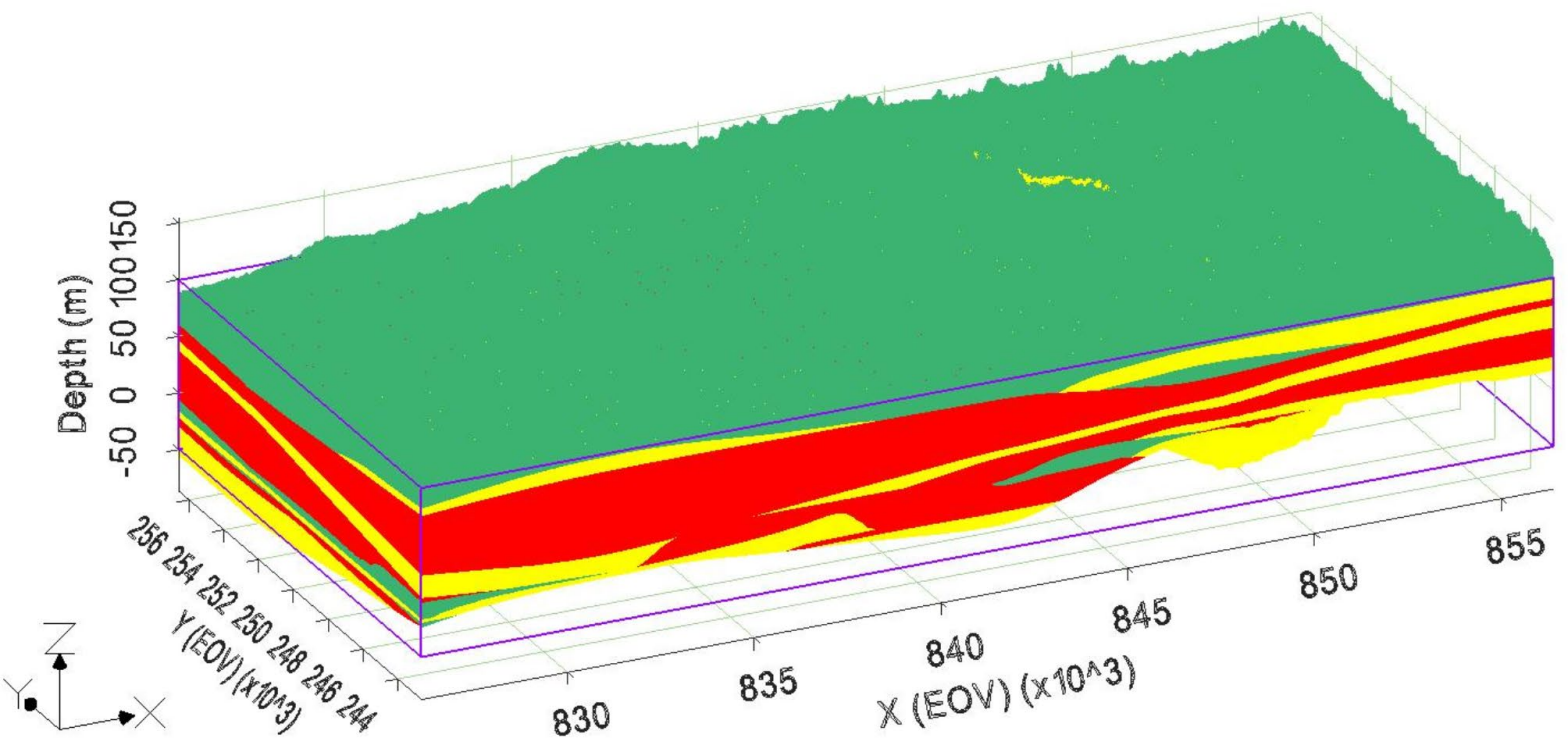
The 3D distribution of the lithological clusters obtained by SOM in which the red color indicates cluster 1 (shale), green to cluster 2 (shaly sand), and yellow to cluster 3 (sand and gravel).

The 1D clustering results along the boreholes were transformed into a 3D geological model (Fig. 13) using solid modules with the groundwater modeling system (GMS) program. The geological frame is composed of 16 layers that are mainly made up of shale, shaly sand, and sand and gravel. During the interpolation process, very thin layers were omitted to provide a clearer representation of the groundwater system. The upper portion of the model, consisting of the CUU and AU, is predominantly composed of shaly sand with thin sandy interlayers^31^. The IVU appears as an elongated sand body, concentrated primarily in the eastern region of the study area, indicating a major aquifer zone^52^. Beneath the valley unit, the LMU is composed mainly of thick layers of shale, forming the deeper and less permeable portion of the groundwater system. These results were in line with the recent investigations conducted by^53^. While the proposed method is effective in sedimentary formations due to the relatively homogenous distribution of petrophysical properties, its applicability may be limited in highly fractured and karst aquifers. These systems exhibit high nonlinearity and anisotropy, making accurate delineation and estimation of petrophysical properties more challenging^76^. For such settings, incorporating additional logs, such as neutron porosity and sonic logs, could significantly enhance the accuracy of characterization. These logs are better suited to account for the complex void spaces and fracture networks prevalent in these aquifer systems.

#### CWT analysis

The CWT analysis was applied to the NGR log to delineate lithological and hydrostratigraphical units within the Quaternary aquifer system. The scalogram analysis of the NGR log, conducted at both small and large scales (S1 and S2 respectively), revealed a distinct separation between shaly and sandy layers based on the wavelet transform coefficients (Fig. 11). At the boundaries between these lithologies, the CWT coefficients were consistently high, indicating sharp transitions between the shale and sand units. High coefficient magnitudes in the scalogram indicate strong correlations between the signal and the wavelet at specific scales and depths, often corresponding to significant lithological boundaries^42^.

At smaller scales, thick shale and sand layers were characterized by uniform, low coefficients, suggesting minimal variation within these homogeneous units. The thinner interbedded shaly and sandy layers exhibited higher coefficients, reflecting greater heterogeneity and sharper lithological transitions within these finer layers. However, the resolution of thin layers at the smaller scales presented challenges in detecting exact boundaries between layers^46^. This limitation is likely due to the difference in the shape of the log signal and the wavelet, which may reduce the precision in identifying sharp transitions in thin layers at coarser resolutions.

At larger scales, the CWT analysis revealed a clear distinction between the major hydrostratigraphical units. Coarsening-upward sequences, composed of intercalated shaly and sandy layers, exhibited mixed wavelet signals. In contrast, the alluvial unit, composed of thick shale layers, displayed uniformly low wavelet coefficients, highlighting the homogeneity of this unit. The boundary between the alluvial unit and the underlying incised valley unit was marked by high coefficients, corresponding to the sharp lithological transition. Within the valley unit, which consists primarily of thick sand layers, lower wavelet coefficients were observed, consistent with the unit’s homogeneous composition.

### Petrophysical and hydrogeological parameters

**Fig. 14:**
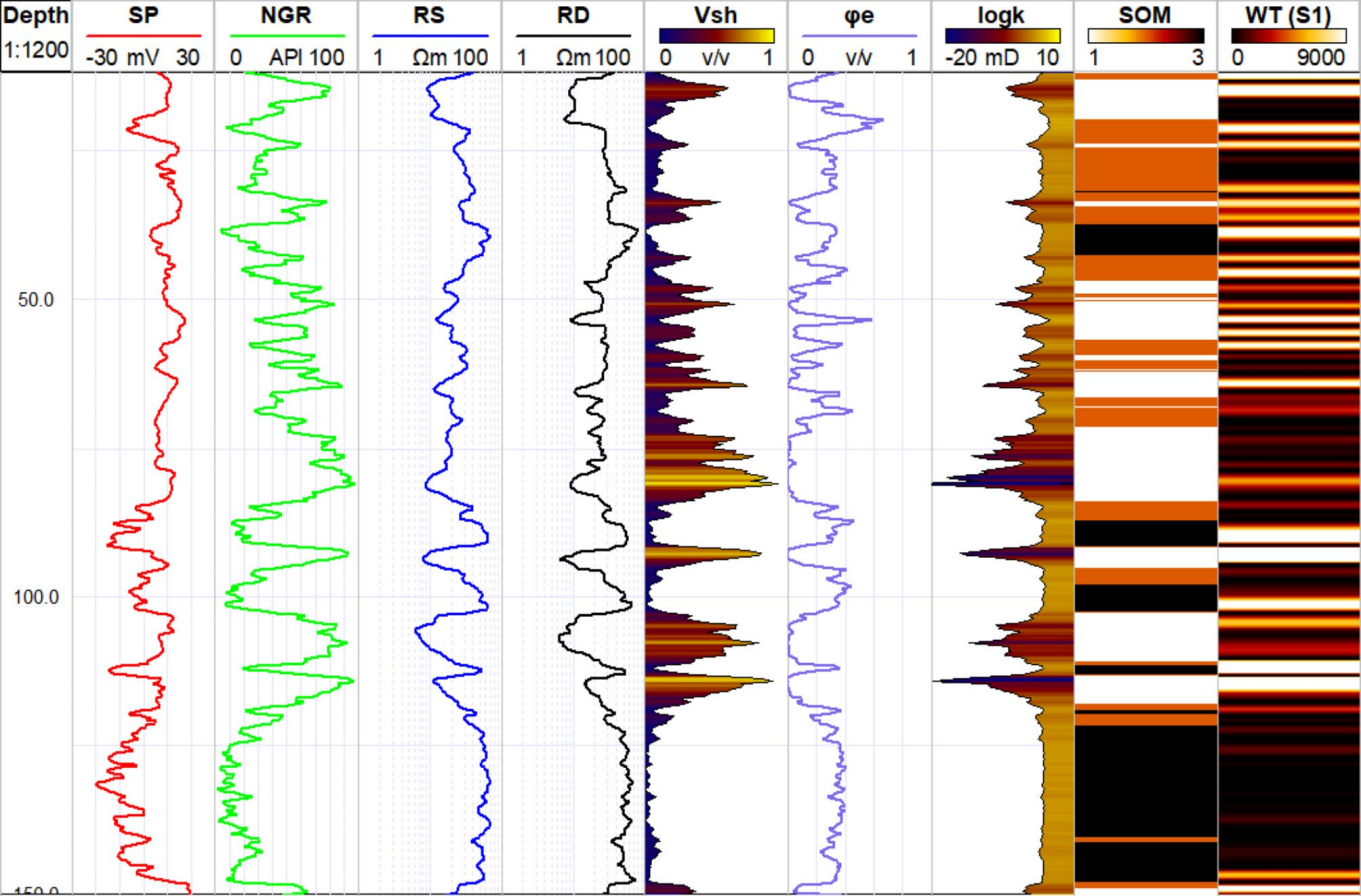
Example of 1D correlation of the hydraulic parameters of shale volume (track 5), effective porosity (track 6), permeability (track 7), SOM results (track 8), and CWT (track 9) in B1 borehole.

**Fig. 15:**
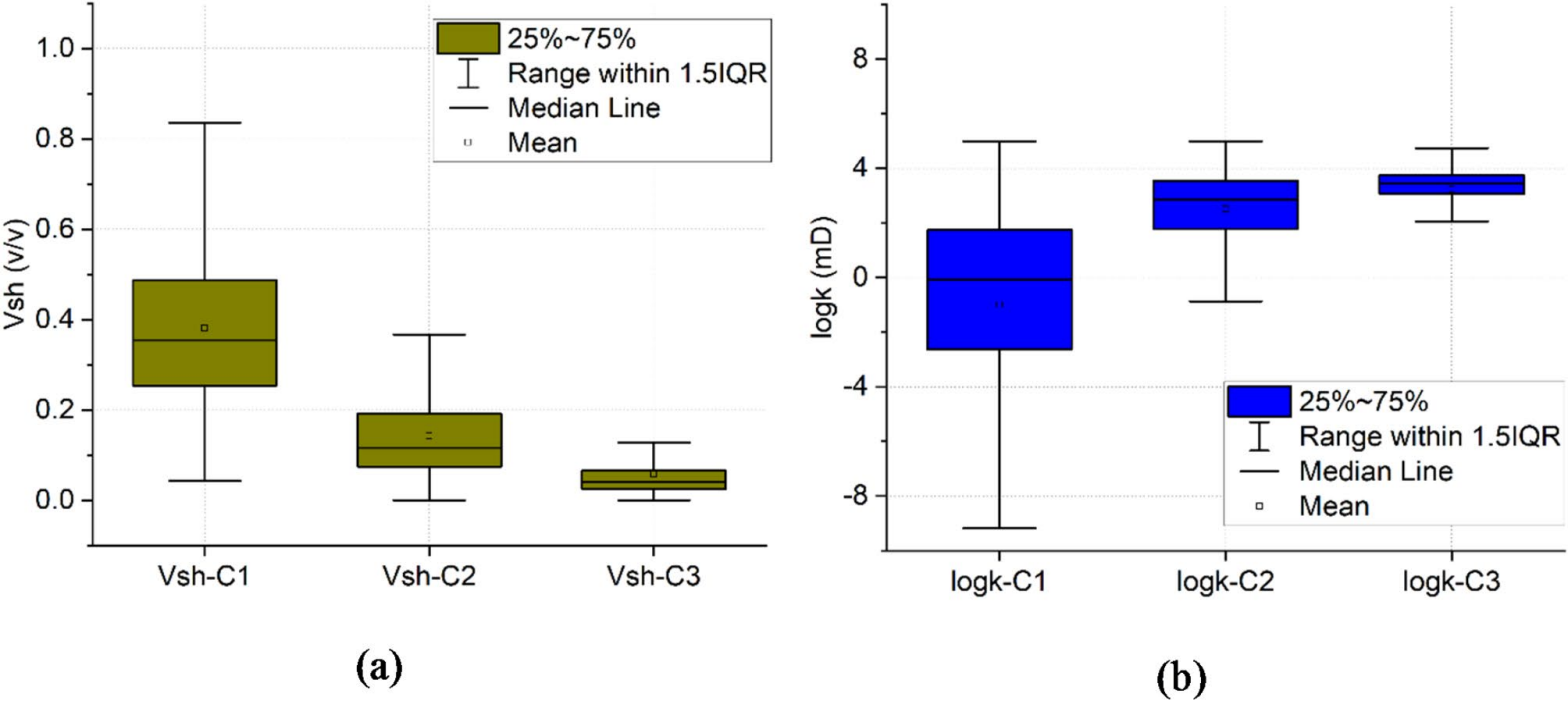
Box plots showing the simple statistics of the estimated (**a**) shale volume and (**b**) permeability for different lithological clusters (C1, C2, and C3).

Following the identification of lithological variation within the groundwater system, Larionov^64^ and Jorgensen^68^ methods were employed to estimate the shale volume and permeability and further confirm the results obtained from SOM analysis. These parameters are determined in 1D along the boreholes (Figs. 14) and 2D across the main hydrostratigraphical units of the groundwater system. The statistical summary of the estimated shale volume and permeability is illustrated in Fig. 15a and b respectively. Cluster 1, characterized by a shale volume ranging from 0.05 to 0.85 with a mean of 0.39, exhibited the lowest permeability values, with a logarithmic permeability range of -9.2 to 5 mD. In contrast, Cluster 2, with a lower shale volume (0.02 to 0.37, mean 0.15), had a higher mean permeability of 2.5 mD. Cluster 3, which had the lowest shale content (0 to 0.12, mean 0.07), showed permeability values ranging between 2 and 4 mD. These findings support the SOM analysis, confirming that clusters with higher shale content correspond to lower permeability.

**Fig. 16:**
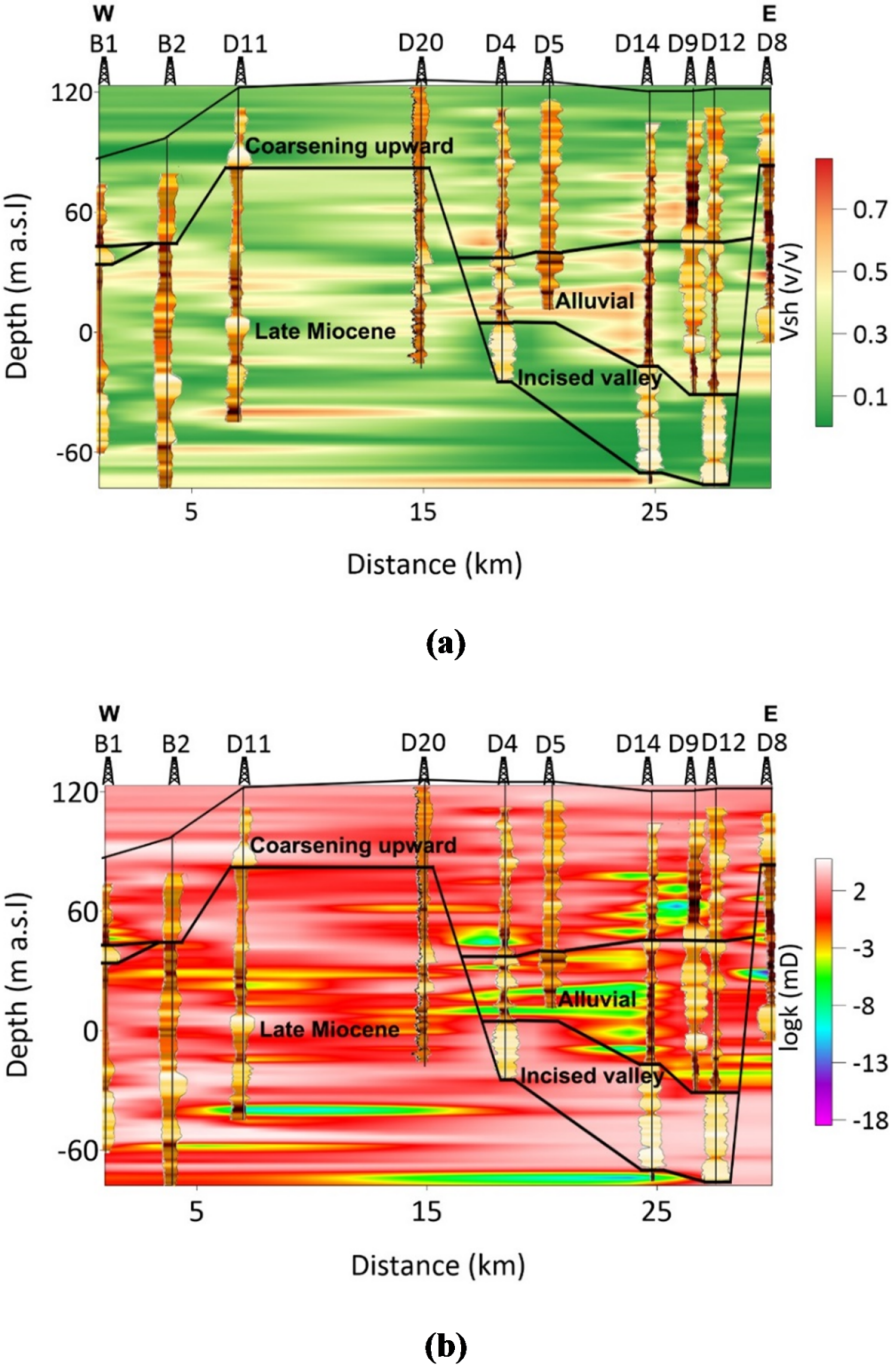
Results of 2D characterization of the main hydrostratigraphical units showing the distribution of (a) shale volume and (b) logarithm of permeability along the geophysical profile.

**Fig. 17:**
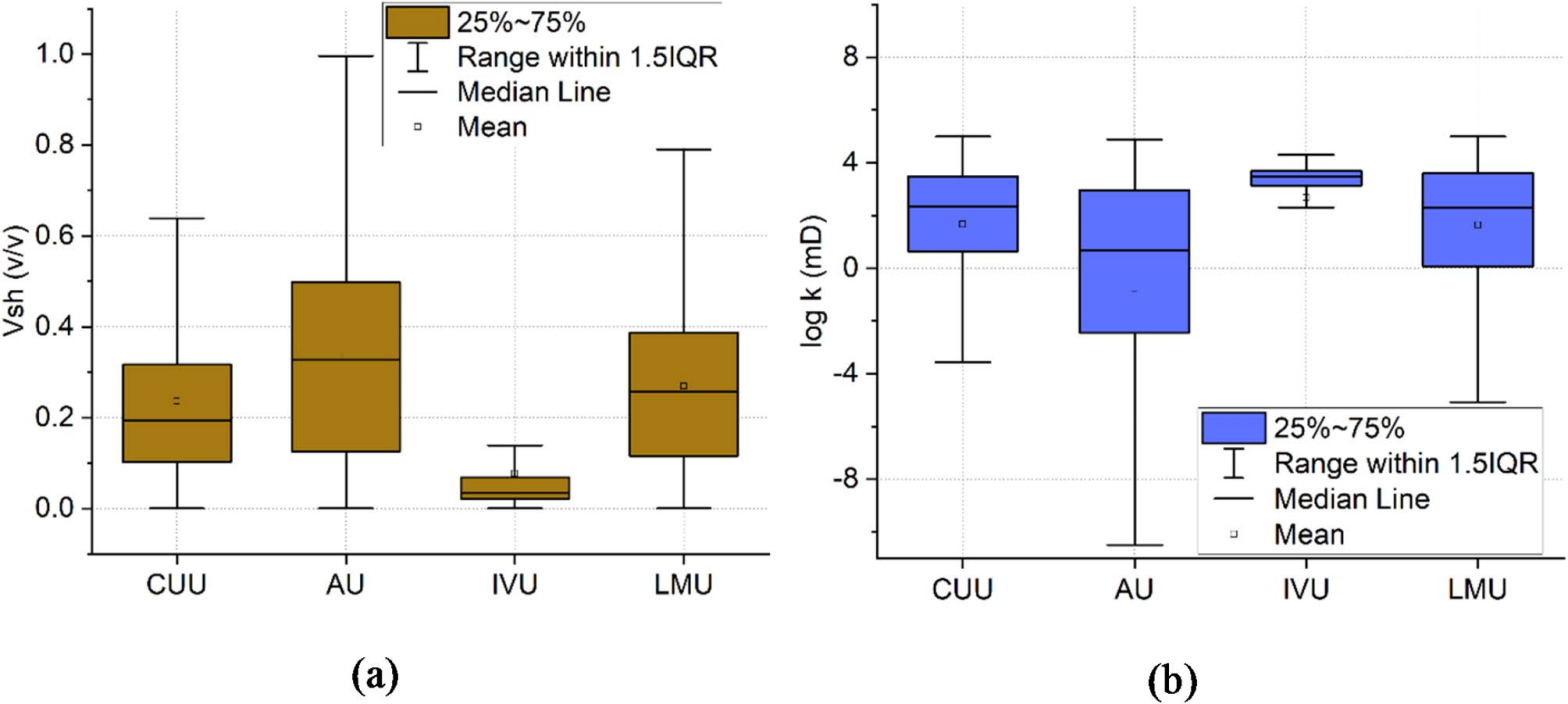
Box plots showing the simple statistics of the estimated (a) shale volume and (b) permeability for main hydrostratigraphical units in the study area.

In the 2D analysis of the aquifer parameters across the hydrostratigraphical units (Fig. 16), distinct characteristics for each unit are indicated. The statistical summary for the estimated shale volume and permeability is illustrated in Fig. 17a and b respectively. The analysis of shale volume and permeability across the main hydrostratigraphical units provides key insights into the lithological and hydraulic characteristics of the groundwater system. In the CUU, shale volume ranged from 0 to 0.64 with a mean of 0.25, while permeability varied between − 3.8 and 5 mD, indicating a relatively balanced shale content and moderate permeability, with a mean value of 1.9 mD. The AU displayed a wider variation in shale volume, ranging from 0 to 0.99 with a mean of 0.33, and lower permeability, averaging − 1.1 mD, reflecting its higher clay content. In contrast, the IVU had the lowest shale content (0 to 0.12, mean 0.05) and the highest permeability, varying between 2 and 4 mD, with a mean of 3 mD, suggesting a more permeable and sandy composition. The LMU showed moderate shale volume (0 to 0.81, mean 0.29) and permeability, ranging from − 5.8 to 5 mD. The results of shale volume and permeability are consistent with the 2D lithological mapping generated by SOM analysis. The AU and LMU were characterized by higher shale content (Cluster 1 and 2), which corresponds to their reduced permeability, making them less favorable for groundwater flow. In contrast, the IVU, identified as consisting predominantly of sandy and gravelly materials (Cluster 3), exhibited low shale content and high permeability. This confirms the SOM result that the IVU represents the most promising groundwater aquifer zone, capable of supporting groundwater flow due to its favorable lithological composition and hydraulic properties^52^.

## Conclusions

This study presents an innovative and integrated approach for the characterization of heterogeneous groundwater systems, combining machine learning techniques and wavelet transform analysis. These methods are applied to the Quaternary aquifer system in the Debrecen area, Eastern Hungary. The key findings and implications of the research are summarized as follows:

- The application of a gated recurrent unit (GRU) neural network for generating synthetic RD logs proved highly effective. The success of the GRU is attributed to its ability to capture complex patterns among the geophysical well logs providing more reliable imputation of the missing data. This preprocessing step significantly improved the quality and consistency of the input data for more robust subsequent analyses.
- The self-organizing map (SOM) analysis is conducted on both unprocessed and preprocessed well logs (GRU-based). The results demonstrated the superiority of the preprocessed data in accurately inferring lithological information. In general, SOM indicated three distinct clusters, which were interpreted as representing shale, shaly sand, and sand and gravel. These clusters aligned with the estimated petrophysical and hydrogeological parameters in which the sandy intervals showed higher permeability and reduced shale content. Accordingly, a 3D geological model is constructed that offers a simplified yet accurate representation of the groundwater system.
- The scalograms of the CWT analysis provided information about the cyclicity of lithological changes and hydrostratigraphical boundaries. CWT revealed clear distinctions between sandy and shaly layers, with formation boundaries marked by high wavelet coefficients. The results of CWT analysis showed close alignment with those obtained from SOM analysis and drilling-derived lithology.
- Building on the findings of this study, future research will focus on adapting and enhancing the proposed methodology for more complex geological settings, such as fractured and karst aquifers. This will involve integrating additional well-logging tools, such as neutron porosity and sonic logs, to better account for the high nonlinearity and anisotropy observed in these systems.
- The analysis of geophysical well logs using machine learning and signal processing techniques demonstrated its potential in hydrogeological applications, offering a cost-effective alternative to traditional methods. The resulting geological model can be used as a basis for building groundwater flow and contaminant transport models leading to effective resource management. The application of this integrated approach can be further applied to different groundwater systems with similar geological settings worldwide.

## Data Availability

The data that support the findings of this study are available from the Supervisory Authority for Regulatory Affairs (SARA), Hungary, but restrictions apply to the availability of these data. Data are, however, available from the corresponding author upon reasonable request and with permission of the Supervisory Authority for Regulatory Affairs (SARA), Hungary.
